# Purification of Fluorescently Derivatized N-Glycans by Magnetic Iron Nanoparticles

**DOI:** 10.3390/nano9101480

**Published:** 2019-10-17

**Authors:** Csaba Váradi, Emőke Sikora, László Vanyorek, Béla Viskolcz

**Affiliations:** Faculty of Materials Science and Engineering, Institute of Chemistry, University of Miskolc, 3515 Miskolc, Hungary; kemsik@uni-miskolc.hu (E.S.); kemvanyi@uni-miskolc.hu (L.V.); bela.viskolcz@uni-miskolc.hu (B.V.)

**Keywords:** glycosylation, magnetic nanoparticles, sample preparation

## Abstract

A novel glycoanalytical approach was developed in this study for the purification of fluorescently derivatized N-glycans. Polyethylene glycol (PEG) modified iron-nanoparticles were synthetized by the combination of sonochemical treatment and combustion method. The prepared nanomaterials were applied for a systematic clean-up optimization to maximize purification efficiency of 2-AA labelled glycans. PEG 1000 modified iron-oxalate was found to be the most effective for the selective enrichment of serum N-glycans providing high reproducibility. Different acetonitrile percentages for binding and washing steps were also tested to ensure the same relative peak areas compared to the unpurified sample. The generated novel clean-up strategy provides a potential route to use in-house synthetized magnetic nanoparticles for glycan sample preparation.

## 1. Introduction

The recognition of protein glycosylation importance in biomarker discovery research and biomanufacturing resulted in the need for novel glycoanalytical platforms [1]. Glycans were found to be involved in several biological events such as cell-cell, cell-matrix interactions, protein folding and stability [2]. Glycosylation of serum proteins is reportedly altered in many inflammatory [3] and malignant diseases [4] moreover in ageing [5], pregnancy [6] and smoking [7] suggesting the need of high-throughput and simplified sample preparation platforms. Glycan profiling is most commonly performed by glycan release, fluorescent labelling, purification and analysis method which can be capillary electrophoresis or high-performance liquid chromatography (HPLC). Liberation of carbohydrate structures from the parent protein is usually completed by PNGase F digestion. After glycan release, fluorescent derivatization is required due to glycans’ lack of fluorophore group [8]. For fluorescent labelling, several compounds are available including 2-anthranilic acid (2-AA) with the main advantage of the possible reductive amination of glycans under aqueous conditions [9]. The derivatization step results in the stoichiometric attachment of a fluorescent tag to each glycan species enhancing detection sensitivity [8]. Once the glycans are fluorescently derivatized, purification is necessitated to remove salts, proteins and excess dye which can influence analytical reliability. Several strategies have been developed in recent years for glycan purification such as solid phase extraction, precipitation, paper chromatography and gel filtration [10]. Most of these methods require sample preconcentration prior to analysis due to the high elution volume of the purification approaches. Magnetic particles have been reportedly efficient to bypass sample preconcentration and simplify the preparative process of APTS (8-Aminopyrene-1,3,6-Trisulfonic Acid)-labelled N-glycans [11]. An important aspect of magnetic particles is that they can be synthetized by the thermal decomposition of iron precursors resulting different iron oxides with magnetic properties. One of the most commonly used iron precursors is iron-oxalate for its unique size distribution, high surface area and magnetization properties [12] although chemical surface modification is often needed to maintain colloidal stability and biocompatibility [13]. With appropriate surface derivatization magnetic iron-oxide nanoparticles (MIONP) can be used for a wide range of applications such as magnetic resonance imaging, detoxification, drug delivery and hyperthermia just to mention a few [14]. The use of MIONPs in glycomics and glycoproteomics applications has been showing increasing tendency in the last few years. Maltose-functionalized hydrophilic iron oxides were found to be efficient for glycopeptide enrichment from complex matrices [15]. Ionic liquid modified hydrophilic MIONPs provided high detection sensitivity and enrichment recovery analyzing Hela exosome glycopeptides. Glutathione-capped iron oxides also provided enhanced detection sensitivity in MALDI-MS glycomics [16]. In this study, polyethylene-glycol (PEG) modified MIONPs were synthetized, characterized and applied for the purification of fluorescently derivatized N-glycans. Targeted sugars were released from human serum by PNGase F digestion followed by 2-AA derivatization. Labelled glycans were purified by PEG 200, 600 and 1000 modified iron-oxalate where PEG1000 provided the highest signal intensity. To minimize potential sample loss, different acetonitrile percentages were also tested for binding and washing steps. The resulted novel clean-up strategy was then applied on 6 individual samples showing excellent reproducibility. Adalimumab and rituximab glycans were also purified by different clean-up methods showing great comparability with conventional purification strategies.

## 2. Materials and Methods

Polyethylene-glycol (200, 600, 1000), acetonitrile, ammonium-hydroxide, acetic acid, formic acid, picoline-borane, 2-aminobenzoic acid (2-AA), and human serum were purchased from Sigma-Aldrich (St. Louis, MO, USA). Iron (II)-oxalate dihydrate (FeC_2_O_4_ ·2H_2_O) was provided by Alfa Aesar (Haverhill, MA, USA). PNGase F was purchased from New England Biolabs (Ipswich, MA, USA). CU (clean-up) cartridges were obtained from Prozyme (Agilent Technologies, Inc. Santa Clara, CA, USA) and normal phase tips were provided by Phynexus (San Jose, CA, USA).

### 2.1. Synthesis Method

Sonochemical treatment and combustion method were combined to synthetize iron oxide nanoparticles. For the synthesis, iron (II)-oxalate dihydrate was used as an iron-precursor, while as dispersant, polyethylene glycols with three different molecular weight (200, 600, 1000), were applied. Thus, 5 g of iron (II)-oxalate was dispersed in 20 g of PEG200, PEG600 and in melted PEG 1000 resulting 3 different mixtures. In the first step, sonochemical treatment was used, where the iron precursor was mixed with the stabilizer (PEG) by a Hielscher UIP1000hdt tip ultrasonic homogenizer (340 W/19.4 kHz) for 10 mins. The homogenization process was followed by 30 min combustion (594 °C based on optical pyrometric measurement by Trotec BP21 pyrometer) using a Bunsen burner resulting the dissociation of PEG coating. Then, the combustion has been ceased and the final product has been cooled down to room temperature.

### 2.2. Characterization Techniques

The size and morphology of magnetic nanoparticles were examined by High-Resolution Transmission Electron Microscopy (HRTEM), FEI Technai G2 high resolution electron microscope, 200 kV (FEI, Hillsboro, USA). The sample preparation was carried by dropping aqueous suspension of the samples on copper grids (Ted Pella Inc., 300 mesh). The crystalline phases composition of the synthetized samples was identified using X-ray diffraction (XRD) Rigaku miniflex II diffractometer, X-ray source: copper anode (λ Cu Kα = 1.5418 Å), I = 15 mA, U = 30 kV (Rigaku Corp., Tokyo, Japan). The surface functional groups were identified by Fourier Transformed Infrared (FTIR) spectroscopy, the spectrums were made in transmission mode, using potassium-bromide pellets with Bruker Vertex 70 instrument (Bruker Corp., Massachusetts, USA). The electro-kinetic (Zeta) potential of the samples was measured in aqueous phase by Malvern Zetasizer, Malvern Nano ZS instrument (Malvern Panalytical Ltd, Malvern, UK) based on laser-doppler electrophoresis.

### 2.3. Glycan Sample Preparation

Glycan release was performed using 5 µL of human serum, 100 µg of rituximab and 100 µg of adalimumab according to the PNGase F digestion protocol of New England Biolabs (Ipswich, MA, USA). The released glycans were labelled by the addition of 10 μL 0.37 M 2-AA and 300 mM picoline borane in 70/30% of dimethyl sulfoxide/acetic acid incubating for 2 h at 65 °C. 

### 2.4. Clean-Up Optimization

Iron-oxalate modified with different molecular weight of PEGs were tested for the selective capture and elution of 2-AA labelled glycans released from human serum. Then, 20 mg of the synthetized nanoparticles were dissolved in 1 mL of HPLC water and 200 µL was transferred to an Eppendorf tube. The nanoparticle containing tube was placed onto a magnetic stand to remove the supernatant. It has been found that 85% of acetonitrile is reportedly efficient for the clean-up of 2-AA labelled glycans thus to compare purification efficiency of PEG200, PEG600 and PEG1000 modified iron-oxalates, and 85% of acetonitrile was used as binding and washing step [17]. Following this, 200 µL of 85% acetonitrile was added to the labelled glycan solution and suspended with the (supernatant removed) nanoparticles while taken off the magnet. After binding, the suspension was placed onto the magnetic stand and the supernatant was removed. This was followed by a washing step using 200 µL 85% acetonitrile suspended with the nanoparticles and removed on the magnet. Finally, the samples were eluted by 100 µL HPLC water from the magnetic nanoparticles and analyzed by ultra-performance hydrophilic interaction liquid chromatography with online fluorescence detection.

### 2.5. LC-FLR Analysis

The prepared N-glycans were analyzed by ultra-performance liquid chromatography equipped with fluorescence detector on a Waters Acquity UPLC instrument under the control of Empower 3 chromatography software (Waters, Milford, MA). Separations were performed by a Waters BEH (Ethylene Bridged Hybrid) Glycan column, 100 × 2.1 mm i.d., 1.7 μm particles, using a linear gradient of 70–55% acetonitrile at 0.4 mL/min in 30 mins, using 50 mM ammonium formate pH 4.4 as mobile phase. Samples were made up in 70% acetonitrile 30% water and 15 μL was injected in all runs. Samples were maintained at 15 °C prior to injection and the separation temperature was 60 °C. The fluorescence detection excitation/emission wavelengths were λ_ex_ = 350 nm and λ_em_ = 425 nm. 

## 3. Results

### 3.1. Magnetic Nanoparticle-Based Glycan Purification

**Figure 1:**
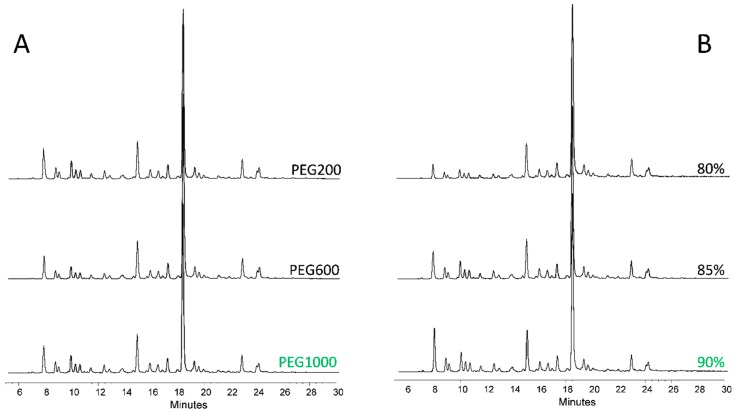
2-AA labelled serum glycan profiles purified by iron-nanoparticles (**A**) and different acetonitrile concentrations (**B**).

**Figure 2:**
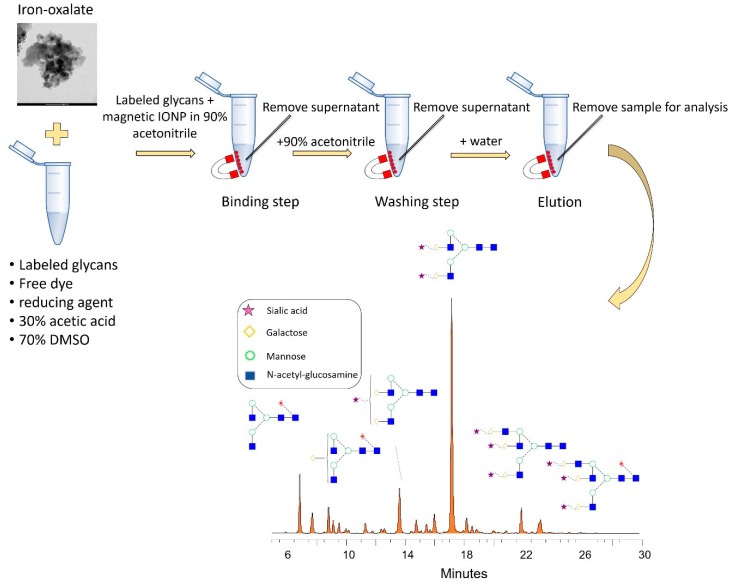
Flow-chart of the magnetic nanoparticle-based sample clean-up.

To obtain high reproducibility and sample recovery, the clean-up optimization was initiated by comparing the 3 synthetized nanoparticles based on the different PEGs. As it shown in Figure 1A, the nanoparticles were found to be suitable for glycan purification. Integrating the relative area percentages, the most similar peak distribution compared to the unpurified sample was provided by the PEG1000 modified nanoparticle thus this was chosen for further experiments. Using this MIONP, different acetonitrile percentages were also investigated for binding and washing steps to maintain the very same relative peak distribution compared to the unpurified sample. As it shown in Figure 1B, the acetonitrile concentration used for glycan purification has a crucial effect on the relative areas especially on neutral glycans (peaks between 7–12 min). Comparing the relative area percentage of the first main peak (retention time 8 min, FA2) to the unpurified sample we have found that using 80% acetonitrile the relative area was 5.58% less, using 85% it was 1.79% less and using 90% it was 0.24% more than the same peak of the unpurified sample indicating the potential sample loss using 80 and 85% acetonitrile. Based on these findings, we have decided to use 90% acetonitrile in all further MIONP-based experiments as the binding and washing step. Combining the advances of PEG1000 iron-oxalate with 90/10 acetonitrile/water as binding and washing, we were able to build up a novel MIONP based clean-up strategy as it shown in Figure 2. The main advantage of this purification method is that in-house synthetized inexpensive materials can be applied for glycan purification, resulting in direct analysis after sample clean-up due to the low elution volume of the MIONP-based clean-up. To examine the reproducibility of this novel nanoparticle-based purification approach, 6 individual serum samples were prepared and analyzed by HILIC-UPLC (ultra performance-hydrophilic interaction liquid chromatography) in triplicates then compared to the unpurified sample. As it shown in Electronic Supplementary Material Table S1, 21 peaks were integrated, and all the 6 replicates showed the same relative distribution compared to the unpurified sample. The reproducibility was calculated based on relative standard deviations of the peak area percentages which was 4.57% on the average of 21 peaks suggesting high reproducibility of the newly developed MIONP based clean-up. The free dye peak of the MIONP purified and unpurified sample was also compared to investigate the efficiency of excess dye removal. As shown in Supplementary Figure S1, most of the free dye was eliminated using our in-house synthetized magnetic nanoparticles suggesting the efficient dye removal. Another critical aspect of sample clean-up is the potential sample loss during the individual binding, washing steps and more importantly the sample recovery in the elution. To ensure that all the glycans are captured in the binding step, no glycans are washed away during washing, and all the captured sugars are eluted, each step was also analyzed as it shown in Supplementary Figure S2. Then, 2-AA labelled glycans from 100 µg of IgG were purified by the developed MIONP-based method (200 µL 20 mg/mL PEG1000) where there were no detectable peaks in any of the binding, washing and 2nd elution steps suggesting a highly efficient sample recovery throughout the purification process. 

**Figure 3:**
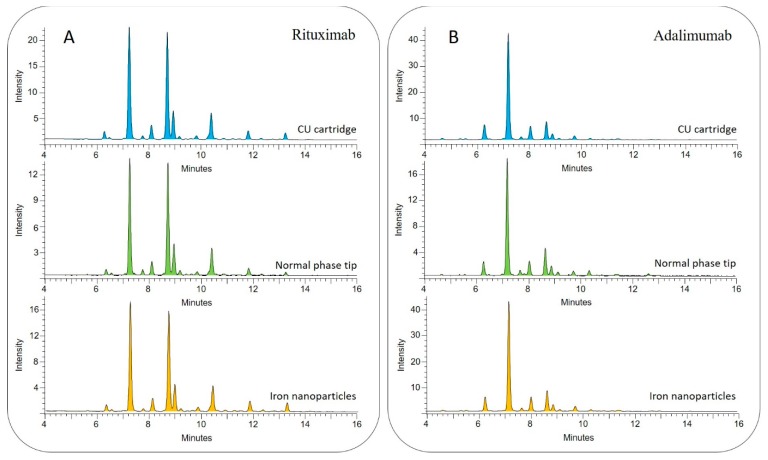
HILIC-UPLC profile of (**A**) rituximab and (**B**) adalimumab glycans purified by traditional methods (blue and green) and in-house synthetized magnetic iron nanoparticles (orange).

Quality control of biopharmaceuticals during monoclonal antibody (mAb) production is critical due to the possible micro heterogeneities carried by the parent proteins such as glycosylation. To examine the compatibility of our MIONP based clean-up strategy in the analysis of mAbs, glycans were released from 100 µg of rituximab and adalimumab and purified by conventional methods and MIONPS after 2-AA labelling. To maintain comparability, for binding, washing and elution steps the very same conditions were applied as in the MIONP-based process. As shown in Figure 3A,B, the obtained profiles showed similar peak distribution across the different methods (CU cartridge, Normal phase tip, MIONP) analyzing both antibodies suggesting great comparability of our newly developed clean-up with traditional methods. These results suggest the potential of the MIONP-based purification method in the analysis of body fluids and biopharmaceuticals as well.

### 3.2. Characterization of PEG1000 Modified Iron-Oxalate

**Figure 4:**
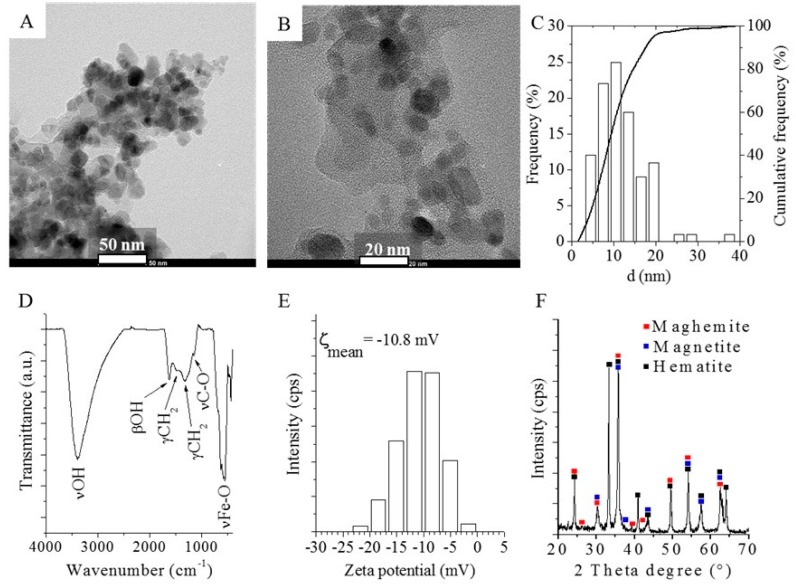
HRTEM images (**A**,**B**) and size distribution (**C**) of the magnetic nanoparticles. Fourier Transformed Infrared (FTIR) spectrum of the iron-oxide surface with the identified functional groups (**D**). Zeta potential distribution of the nanoparticles in aqueous phase (**E**) and X-ray diffraction (XRD) pattern of the iron-oxide sample (**F**).

To understand the origin of glycan capture capability and magnetic susceptibility, the nanoparticles were characterized by multiple techniques. The size distribution was measured based on the HRTEM images where the diameters were found between 3.3 and 36.9 nm while 97% of them were smaller than 20 nm (Figure 4A–C). Nanoparticle surface was covered by -OH, Fe-O and hydrocarbon functional groups characterized by FTIR spectroscopy. The -OH group was identified by OH stretching (νOH), bending (βOH) and C-O stretching (νCO) at 3401.6 cm^−^^1^, 1624 cm^−^^1^ and 1149.2 cm^−^^1^ respectively. The band of the νFe-O vibrational mode was located at 562 cm^−^^1^ and the surface hydrocarbon presence was confirmed by γCH2 bands. Fe-O and C-O bonds were also found probably due to the partial oxidation of the nanoparticle surface during the preparation. Also, 7.7% carbon content was identified by element analysis which was in oxidized form originating from the thermal decomposition of PEG molecules during combustion. The proton dissociation of the hydroxyl groups provided negative zeta potential (electro-kinetic potential with Boltzmann like distribution until 25 mV with an average of 10.8 mV. The iron-oxide phases were identified as three types of FeO particles, namely maghemite, magnetite and hematite (Figure 4F) with the relative distribution of 10.97 wt% of magnetite (Fe_3_O_4_), 55.58 wt% of hematite (α-Fe_2_O_3_) and 33.45 wt% of maghemite (γ-Fe_2_O_3_), providing the magnetic properties to the synthetized material. The contact angle was found to be ~5° suggesting extremely high hydrophilicity of the synthetized nanoparticles (Supplementary Figure S3). Hydrophilic surfaces are reportedly able to adsorb carbohydrates applying a polar mobile phase (water) in a high content of organic solvent (acetonitrile) [18]. The possible mechanisms of organic material adsorption on iron oxides are also described by Gu et al. involving ion exchange, surface complexation, hydrogen bonding and cation bridging [19]. Iron oxides are reportedly able to form hydrogen donor interactions with hydroxyl groups and adsorb polar compounds [20]. Glycans exhibit numerous hydroxyl groups providing the potential of hydrogen bond formation with iron oxides. This is supported by the fact that the acetonitrile concentration for binding and washing steps had to be increased (water content decreased) thus iron oxides formed hydrogen donor interaction with the OH group of glycans instead of water. The addition of water as an eluent resulted in the dissociation of hydrogen bonds, thus the adsorbed sugars could be isolated from supernatant.

## 4. Conclusions

Magnetic iron-oxide nanoparticles with hydrophilic surface characteristics were synthetized in this study for the selective enrichment of fluorescently derivatized N-glycans. PEG1000 modified iron oxalate was found to be the most effective to capture and purify the targeted sugars. The optimal acetonitrile concentration for binding and washing steps was also investigated resulting in high reproducibility and sample recovery. The developed purification approach was compared to conventional methods suggesting great comparability with other clean-up strategies. This novel method provides a potential recipe to synthetize magnetic nanoparticles and their application for glycan sample preparation allowing low elution volumes thus direct analysis after sample clean-up. 

## Supplementary Materials

The following are available online at https://www.mdpi.com/2079-4991/9/10/1480/s1, Table S1: Relative area distribution of 2-AA labelled serum glycans purified by PEG1000 modified iron-oxalate and unpurified sample. Figure S1: Free dye peak area comparison of unpurified and IONP purified sample, Figure S2: Analysis of binding, washing, 1st and 2nd elution steps to investigate purification efficiency of MIONP based clean-up suggesting maximized sample recovery, Figure S3: Contact angle assay of the synthetized nanoparticles (Iron-oxalate/PEG1000).
