# Supplementary material for: Purification of Fluorescently Derivatized N-Glycans by Magnetic Iron Nanoparticles
Source: Nanomaterials (Basel). 2019 Oct 17;9(10):1480. doi: 10.3390/nano9101480 (PMC6835309; doi:10.3390/nano9101480)
Supplement: Supplementary file 1 [file nanomaterials-09-01480-s001.pdf]

Supplementary Material

Purification of Fluorescently Derivatized N-Glycans by Magnetic Iron Nanoparticles

Csaba Váradi *, Emőke Sikora, László Vanyorek and Béla Viskolcz

Faculty of Materials Science and Engineering, Institute of Chemistry, University of Miskolc, Miskolc 3515 Hungary; kemsik@uni-miskolc.hu (E.S.); kemvanyi@uni-miskolc.hu (L.V.); bela.viskolcz@uni-miskolc.hu (B.V.)

* Correspondence: kemcsv@uni-miskolc.hu; Tel.: +36308947730

Table S1: Relative area distribution of 2-AA labelled serum glycans purified by PEG1000 modified iron-oxalate and unpurified sample.

	**Unpurified**	**Purification1**	**Purification2**	**Purification3**	**Purification4**	**Purification5**	**Purification6**	**Average**	**Stdev**	**RSD%**
Peak#1	6.52	6.44	6.89	6.38	6.56	6.62	7.21	6.68	0.31	4.68
Peak#2	1.69	1.73	1.82	1.71	1.75	1.76	1.92	1.78	0.08	4.43
Peak#3	1.44	1.41	1.51	1.42	1.46	1.49	1.58	1.48	0.06	4.27
Peak#4	3.17	2.93	3.03	2.90	3.01	3.03	3.24	3.02	0.12	3.93
Peak#5	1.67	1.64	1.65	1.57	1.65	1.67	1.78	1.66	0.07	4.06
Peak#6	1.39	1.40	1.44	1.35	1.42	1.41	1.52	1.42	0.06	4.07
Peak#7	1.3	1.51	1.43	1.40	1.66	1.54	1.62	1.53	0.10	6.71
Peak#8	1.54	1.36	1.34	1.28	1.42	1.38	1.43	1.37	0.06	4.03
Peak#9	1.09	1.01	0.95	0.99	1.10	1.07	1.11	1.04	0.07	6.41
Peak#10	1.96	1.96	1.99	1.89	2.07	1.99	2.09	2.00	0.07	3.57
Peak#11	8.02	8.96	8.54	8.86	8.93	9.06	9.17	8.92	0.21	2.39
Peak#12	2.77	3.03	3.04	2.76	2.95	2.87	3.13	2.96	0.13	4.46
Peak#13	2.54	2.52	2.52	2.34	2.46	2.39	2.54	2.46	0.08	3.30
Peak#14	3.6	3.66	3.69	3.63	3.62	3.63	3.68	3.65	0.03	0.74
Peak#15	1.31	1.19	1.32	1.13	1.14	1.10	1.24	1.19	0.08	6.89
Peak#16	43.25	42.57	42.37	43.87	42.94	43.44	41.68	42.81	0.78	1.83
Peak#17	4.8	4.38	4.59	4.09	4.17	3.92	4.13	4.22	0.23	5.57
Peak#18	1.74	1.65	1.67	1.44	1.43	1.40	1.51	1.52	0.12	7.60
Peak#19	5.32	5.96	5.60	5.98	5.61	5.55	5.17	5.65	0.30	5.32
Peak#20	1.65	1.62	1.57	1.79	1.69	1.72	1.53	1.65	0.10	5.95
Peak#21	3.22	3.05	3.06	3.23	2.97	2.95	2.70	2.99	0.17	5.76

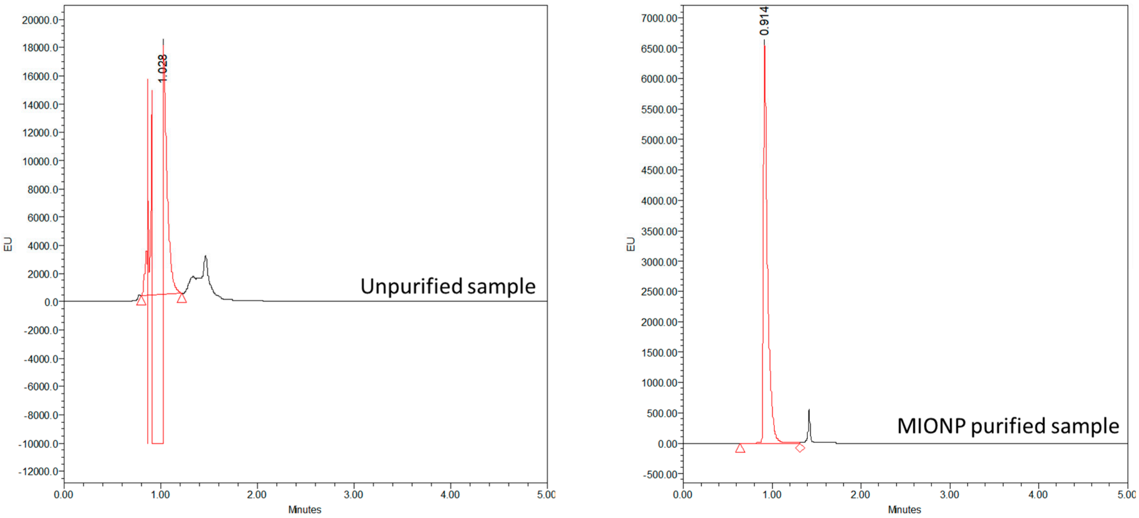

Figure S1: Free dye peak area comparison of unpurified and IONP purified sample.

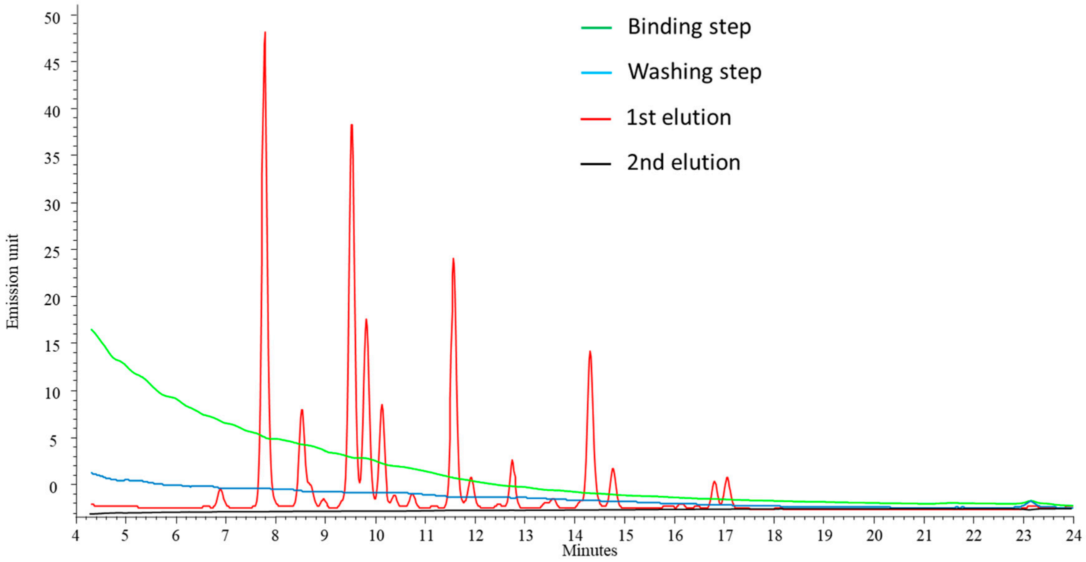

Figure S2: Analysis of binding, washing, 1st and 2nd elution steps to investigate purification efficiency of MIONP based clean-up suggesting maximized sample recovery.

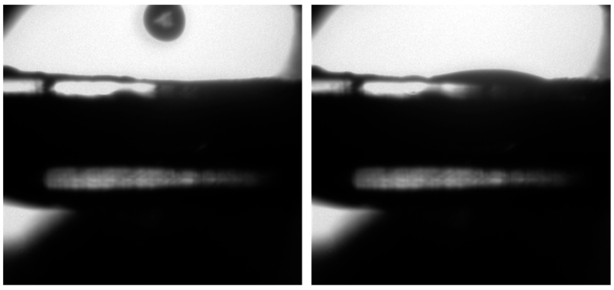

Figure S3: Contact angle assay of the synthetized nanoparticles (Iron-oxalate/PEG1000).
